# Effectiveness of extended shutdown measures during the ´Bundesnotbremse´ introduced in the third SARS-CoV-2 wave in Germany

**DOI:** 10.1007/s15010-021-01713-7

**Published:** 2021-10-20

**Authors:** Andreas Schuppert, Katja Polotzek, Jens Karschau, Christian Karagiannidis

**Affiliations:** 1 Institute for Computational Biomedicine, JRC for Computational Biomedicine, RWTH Aachen University, University Hospital Aachen, Aachen, Germany; 2 Centre for Evidence-Based Healthcare, University Hospital Carl Gustav Carus and Carl Gustav Carus Faculty of Medicine, Technische Universität Dresden, Dresden, Germany; 3 Department of Pneumology and Critical Care Medicine, Cologne-Merheim Hospital, ARDS and ECMO Centre, Kliniken Der Stadt Köln, Witten/Herdecke University Hospital, Cologne, Germany

**Keywords:** COVID, SARS-CoV-2, Shutdown, Spreading, Germany

## Abstract

A third SARS-CoV-2 infection wave has affected Germany from March 2021 until April 24th, until the ´Bundesnotbremse´ introduced nationwide shutdown measures. The ´Bundesnotbremse´ is the technical term which was used by the German government to describe nationwide shutdown measures to control the rising infection numbers. These measures included mainly contact restrictions on several level. This study investigates which effects locally dispersed pre- and post-´Bundesnotbremse´ measures had on the infection dynamics. We analyzed the variability and strength of the rates of the changes of weekly case numbers considering different regions, age groups, and contact restrictions. Regionally diverse measures slowed the rate of weekly increase by about 50% and about 75% in regions with stronger contact restrictions. The 'Bundesnotbremse' induced a coherent reduction of infection numbers across all German federal states and age groups throughout May 2021. The coherence of the infection dynamics after the 'Bundesnotbremse' indicates that these stronger measures induced the decrease of infection numbers. The regionally diverse non-pharmaceutical interventions before could only decelerate further spreading, but not prevent it alone.

**Supplementary Information:**

The online version contains supplementary material available at 10.1007/s15010-021-01713-7.

## Diverse rise of infection dynamics throughout Germany

Germany was affected by a strong third SARS-CoV-2 wave related to the emerging alpha variant in spring 2021 [1, 2]. The spreading dynamics showed reduced case numbers by April for multiple reasons. The German vaccination program beginning in the end of December 2020 initially focused on highly vulnerable groups [3], in particular, the elderly [4, 5]. Most of the population has, thus, not yet been immunized and non-pharmaceutical interventions (NPI) remained the key approach for spreading control [5–8].

Analyses of NPI efficiency for breaking SARS-CoV-2 infection dynamic is important but hampered by the interaction of a multitude of impacts beside vaccination such as seasonality, test strategies, anticipation, and heterogeneous bundles of NPIs set in place locally [9]. Moreover, the efficacy of NPIs depends on the context of the dominating mode of spreading, for example nearly linear or exponential growth in infection dynamics [10]. Hence, analyses of NPI efficiency can hardly be performed by statistical analysis of overall dynamics without deconvolution of the respective effects of diverse measures.

In our previous analysis [10], we assessed NPIs in the context of spreading by different patterns of coherent infection dynamics across the sixteen federal states in Germany and different age groups.

The local NPIs implemented during the raise of the third wave in Germany greatly differed regionally (Table 1). The aim of the current work was, therefore, to determine the effects of the extended shutdown set in place by April 24, 2021, by comparing the infection dynamics before and after this date across the federal states and age groups. These measures, also called ´Bundesnotbremse´ (verbatim ´federal emergency break´), induced nationwide control of SARS-CoV-2 infection dynamics by introducing consistent contact reductions across all states and were associated with an ongoing reduction of daily new cases. The individual contact reductions of the “Bundesnotbremse” had been established beforehand in some federal states allowing us to study the effect of coherent measures in contrast to local measures on control of nation-wide pandemic spreading.

**Table 1:** Prior to the ´Bundesnotbremse´ across the German federal states a variety of different contact restrictions was in place depending on the recorded seven-day incidence per 100,000 inhabitants (* 7day/100 k) within the state

Contact restrictions in the German federal states	Incidence < 100 * 7day/100 k		Incidence ≥ 100 * 7day/100 k	
	< 5 persons	≥ 5 persons	< 5 persons	≥ 5 persons
Baden-Württemberg		x		x
Bayern		x	x	
Berlin	x		x	
Brandenburg		x	x	
Bremen		x		x
Hamburg	x		x	
Hessen		x		x
Mecklenburg-Vorpommern		x		x
Niedersachsen	x		x	
Nordrhein-Westfalen		x		x
Rheinland-Pfalz		x	x	
Saarland		x		x
Sachsen		x		x
Sachsen-Anhalt		x	x	
Schleswig-Holstein		x	x	
Thüringen		x		x

The above number of allowed contacts serves a one example of the diversity of non-pharmaceutical interventions throughout Germany (Suppl. Figure 2). Different states implemented stricter regulations, such as contact restrictions regarding different households or numbers of persons, or curfews at night-time across the entire state, each only in hot spot counties or across the entire state

## Regional and age-stratified analysis of the infection dynamics

**Fig. 1:**
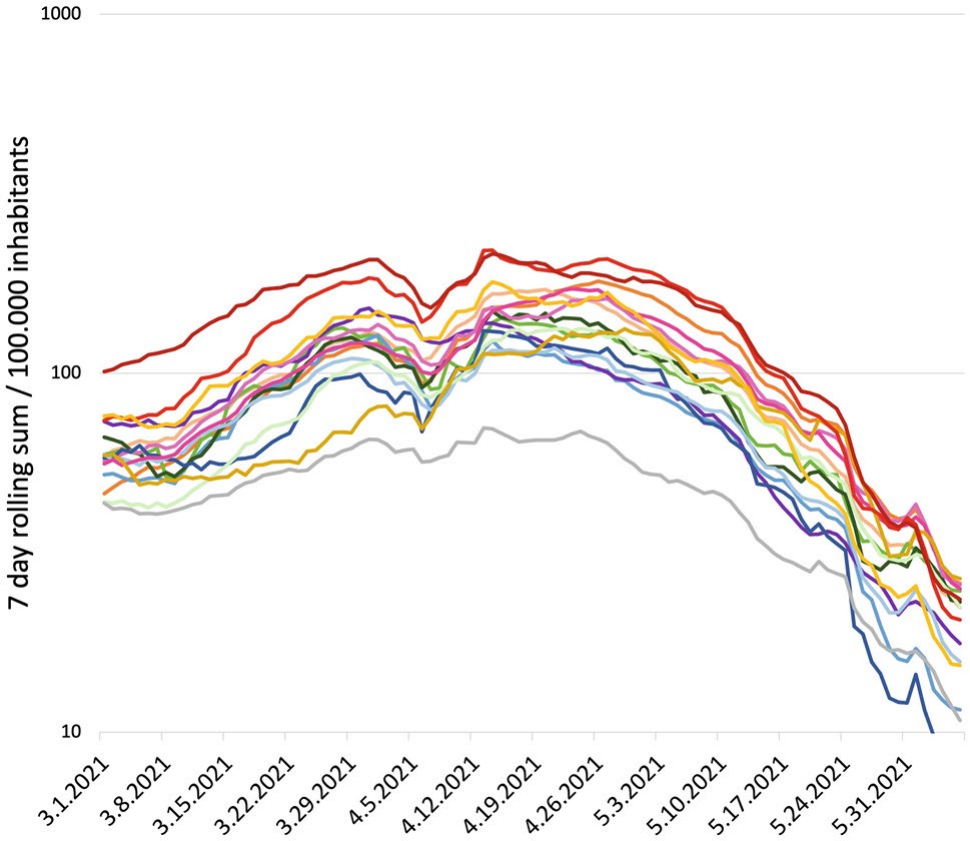
The 7-day incidence per 100.000 inhabitants for the 16 German federal states depicts three different phases during the third wave in Germany. Prior to spring break 2021 we observed very different dynamics among the states, which harmonized to a similar rate of reduction across all states after unified NPIs throughout Germany starting from end of April 2021. The linear slopes in semi-logarithmic scaling emphasize the exponential behavior. Further, first the slopes are different and disordered, later they coincide and keep their order. (See Suppl. Figure 3 for color code.)

For our analysis, we used the daily reports on age-federal state stratified incidences from the RKI Dashboard [1]. We split the third wave into three different phases, separated by well-defined events: exponential growth during March 2021 before the Easter holidays, slowing down during the Easter period and a reduction phase after initiation of the “Bundesnotbremse”. The linear shape of the case numbers in semi-logarithmic scaling (Fig. 1) during the rising phase visualizes exponential growth, where the different slopes correspond to different effective reproduction numbers. The data around spring break are strongly influenced by testing and reporting issues around the Easter holidays with huge reporting gaps. Hence, we considered the mean slopes between beginning and end of the Easter holidays for our analysis (Fig. 1). As in parallel to NPI’s, by March the majority of those ones aged over 80 years were vaccinated, we focused our attention to the age groups between 35 and 79 years [5].

For suppression of the weekly anomalies of infection incidence data due to non-continuous reporting in Germany, we use moving 7-day means of case numbers for six age groups across all 16 federal states, normalized to 100.000 inhabitants within each age group. To compare absolute change rates per day, we use finite differences from the growth phase interval March 17–March 29, the slowing down phase interval March 29–April 24 and the reduction phase interval April 24–May 14, 2021. Within each interval we calculated the mean growth rate per day for each federal state and age group using the respective 7-day means resulting in the mean change rate as described above. For assessment of the impact of local contact restrictions set in place before Easter period, we used the highly diverse regulations for private contacts in case of 7-day incidences below and above 100 7d/100.000 inhabitants as a marker for the overall strength of local restrictions set in place before and within slowing down phase (Table 1).

## The effects of NPIs before and after the ´Bundesnotbremse´

**Fig. 2:**
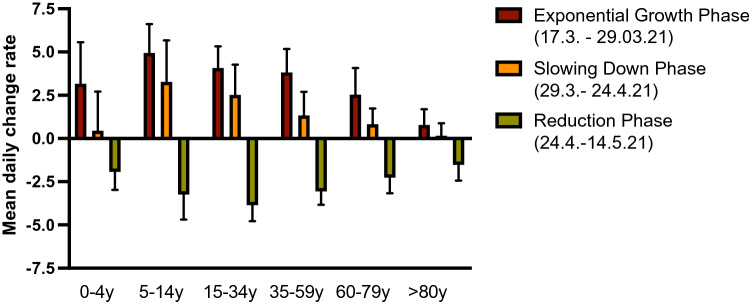
The mean of the daily rate of change of the 7-day incidences among all German states (bar height) was positive across all age groups prior to the ´Bundesnotbremse´ and negative afterwards. The low rate of the elderly reflects the immunization among this population group that was prioritized in the beginning of the German vaccination program. In the reduction phase we found less variance (thin lines, measured by standard error of the mean) across the states than in the slowing down phase characterized by various NPIs

We found that the dynamics of the growth phase has been reduced throughout the slowing down phase for all age groups, followed by a remarkable downturn in the reduction phase (Fig. 2). However, the p values for the difference of change rates between growth phase and slowing down phase as well as slowing down phase and reduction phase show clear significance for the observed dynamics, stratified by age groups (Suppl. Figure 1A, Wilcoxon test) and even when stratified by federal states who differed in testing strategies for children (Suppl. Figure 1B, two-sided I test). Notably, the decrease of dynamics after the ´Bundesnotbremse´ exceeds by far the decrease induced by the slowing down phase before. An exception is the state of Hamburg, where the decrease induced by the ´Bundesnotbremse´ is not significant (*p* = 0.98). Hamburg, however, had installed hard contact restrictions, including a curfew, three weeks prior to the ´Bundesnotbremse´ explaining the insignificant effect of the ´Bundesnotbremse´ in Hamburg.

**Fig. 3:**
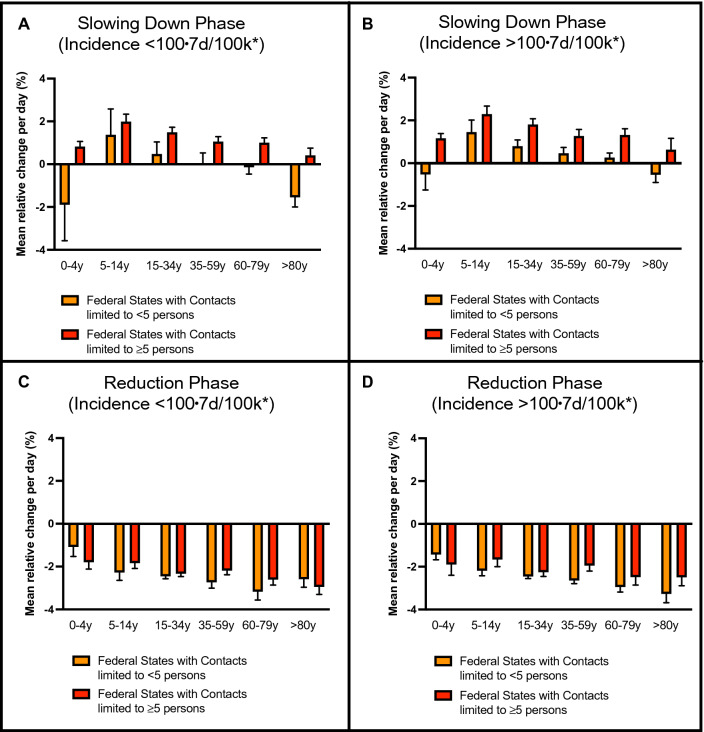
During the period of heterogeneous NPIs (Suppl. Table 1) the mean daily relative change per age group differed among the states (Suppl. Figure 1). Those states with a less than five allowed contacts already at 7-day incidences per 100.000 inhabitants (* 7day/100 k) below 100 exhibited slower rates of increase (**A**). So did states with a stronger reduction of contacts when 100 was reached (**B**). During the reduction phase after the ´Bundesnotbremse´ we observed similar negative relative changes across all states with decreased standard error of the mean (thin lines) compared to the mean (bar heights). Those states with early contact reduction set in place experienced slightly faster decreased (**B**, **C**)

A significant decrease of infection dynamics has started around the Easter period, about three weeks before the ´Bundesnotbremse´. To assess the potential impact of NPI’s on the slowing down phase, we analyzed the daily growth dynamics within the slowing down phase in the federal states with moderate to strong contact restrictions before homogenization by the ´Bundesnotbremse´ (Fig. 3). Remarkably, the daily growth rates within the slowing down phase have been significantly higher in federal states with moderate contact regulations (red bars) compared to federal states with strong contact regulations (orange bars) (Fig. 3A, B). This difference disappeared after the ´Bundesnotbremse´ where we found strong reduction of incidence independent of prior local regulations (Fig. 3C, D). These observations are confirmed by statistical significance testing showing highly significant correspondence of hard contact regulations and slowing down of infection dynamics (Suppl. Figure 2). We could not find any significant correspondence between the contact regulations and infection dynamics in the growth and reduction phases.

## The impact of the ´Bundesnotbremse´

Our findings on spatially age stratified analyses of the third wave in Germany show remarkable differences in infection dynamics confirming our earlier results concerning the second wave. The infection dynamics after the ´Bundesnotbremse´ were characterized by a steep, coherent decline. In contrast, the slowing down phase before the ´Bundesnotbremse´ resulting from school holidays, diverse contact restrictions during Easter period and also a changing behavior of the population showed only a slight and inhomogeneous decrease of infection spreading. As vaccination made rapid progress primarily within the age group 80 + and age group 60–79 years, the infection dynamics differences might be driven by vaccination progress. Moreover, testing strategies have been changed within the period for preschool and schoolchildren, such that data for the age groups below 14 years may be affected by changes in testing strategies, less by NPI’s. Remarkably, this decline was highly significant in age groups between 15 and 59, where vaccination had minor impact within this timeframe. Within the timespan of our analysis in April and May 2021 weather has been extraordinarily cold and wet with similarly cold temperatures in April and May [11, 12]. Hence we believe that effects of seasonality did not contribute significantly to the observed nationwide decline of the infections in May compared to April 2021.

Moreover, the highly significant correlation between strong contact regulations set in place locally before the ´Bundesnotbremse´ indicates that even the modest decrease of infection growth in slowing down phase was induced by NPI’s. This effect was enforced nationwide by the ´Bundesnotbremse´ indicating a significant impact of NPI’s on breaking the third infection wave throughout Germany. As coherent decrease of infection dynamics may reduce the chance for local outbreaks, the homogenization of measures by the ´Bundesnotbremse´ may have contributed significantly to the successful management of the third wave in Germany.

## Supplementary Information

Below is the link to the electronic supplementary material.

Supplementary file1 (DOCX 64 kb)
