# Supplementary material for: Effectiveness of extended shutdown measures during the ´Bundesnotbremse´ introduced in the third SARS-CoV-2 wave in Germany
Source: Infection. 2021 Oct 20;49(6):1331–5. doi: 10.1007/s15010-021-01713-7 (PMC8526993; doi:10.1007/s15010-021-01713-7)
Supplement: Supplementary file 1 — Supplementary file1 (DOCX 64 kb) [file 15010_2021_1713_MOESM1_ESM.docx]

**Effectiveness of extended shutdown measures during the ´Bundesnotbremse´ introduced in the third SARS-CoV-2 wave in Germany**

**Supplementary material**

Andreas Schuppert^1*^, Katja Polotzek^2*^, Jens Karschau^2^ and Christian Karagiannidis^3^

^1^ Institute for Computational Biomedicine, JRC for Computational Biomedicine

RWTH Aachen University, University Hospital Aachen

^2^ Centre for Evidence-based Healthcare, University Hospital Carl Gustav Carus and Carl Gustav Carus Faculty of Medicine, Technische Universität Dresden

^3^ Department of Pneumology and Critical Care Medicine, Cologne-Merheim Hospital, ARDS and ECMO centre, Kliniken der Stadt Köln, Witten/Herdecke University Hospital, Cologne, Germany

**Supplemental Figure 1**

**A.**

**B.**

**Supplemental Figure 1 A and B:** The Decrease of the infection dynamics after the „Bundesnotbremse“ exhibits high significance (p<.001 for all age groups, Wilcoxon test (fig. 1a), 2-sided t-test (Fig 1b) due to small country-wise stratified data samples), remarkably more significant than the decrease between growth and slowing down phase.

**Supplementary Figure 2**

**Supplemental Figure 2:** The infection dynamics in the slowing down phase in federal states with string contact regulations before the ´Bundesnotbremse´ (less than five contacts) exhibits significantly (p<.001) lower growth compared to federal states allowing five or more contacts. No significance is found for exponential the growth phase, where almost no contact regulations have been set in place and for the reduction phase, where the ´Bundesnotbremse´ had homogenized all differences. The bars show |log10(p)| with added negative sign if the dynamics within states with strong regulations is below the dynamics in states with moderate regulations, and positive sign otherwise.

**Supplemental Figure 3**

7-day incidence per 100.000 inhabitants for the 16 German federal states together with legend color code.
